# Prospective Clinical Study of Postoperative Individualized Adjuvant Chemotherapy for Patients with Non-Small-Cell Lung Cancer Based on mRNA Expression of the Molecular Markers *RRM*1, *TUBB*3, and *ERCC*1

**DOI:** 10.1155/2021/8820691

**Published:** 2021-09-23

**Authors:** Jingyao Li, Yang Qiu, Junxiu Yi, Xi Liu, Shixin Zhang, Deli Tan, Tao Jing, Yi Liao, Meng Tang, Jie Liu, Haidong Wang

**Affiliations:** ^1^Department of Thoracic Surgery, Southwest Hospital, Army Medical University, Chongqing 400038, China; ^2^Department of Ultrasonic Diagnosis, Southwest Hospital, Army Medical University, Chongqing 400038, China; ^3^Department of Vasculocardiology, Southwest Hospital, Army Medical University, Chongqing 400038, China

## Abstract

**Objective:**

To investigate the clinical significance of the mRNA expression of *RRM*1, *TUBB*3, and *ERCC*1 in non-small-cell lung cancer (NSCLC) tissues for the selection of adjuvant/postoperative chemotherapy regimens.

**Methods:**

Patients diagnosed with stage Ib-IIIa NSCLC were enrolled and randomly divided into a control group (undetected group) and an experimental group (detected group) after radical operation. The control group randomly received chemotherapy with gemcitabine plus cisplatin or paclitaxel plus cisplatin. The mRNA expression of *RRM*1, *TUBB*3, and *ERCC*1 was detected in the experimental group before chemotherapy, and based on the detected expression, the chemotherapy regimen of cisplatin plus gemcitabine or cisplatin plus paclitaxel was chosen. The disease-free survival (DFS) of the control group and experimental group was compared.

**Results:**

Pathological type, stage, gene expression detection, and treatment method were not significantly correlated with DFS (*P* > 0.05). In the subgroups treated with gemcitabine, the median DFS was 17 months in the detected group and 10.5 months in the undetected group (hazard ratio = 0.2147, 95% confidence interval: 0.07909–0.5827, *P*=0.0025). Multivariate regression analysis was performed to analyse whether gene expression detection was independently correlated with DFS in the subgroups treated with gemcitabine (*P*=0.025). In the detected group, the prognosis of patients with low expression of *RRM*1 was better than that of patients with high expression of *RRM*1 after paclitaxel treatment (*P*=0.0039).

**Conclusions:**

The selection of chemotherapy regimen based on mRNA expression of the *RRM*1, *TUBB*3, and *ERCC*1 genes may improve selection of candidate patients to receive clinical chemotherapy. However, large-scale prospective clinical studies are needed for in-depth investigation.

## 1. Introduction

Lung cancer is currently the leading cause of cancer-related death in humans, and its morbidity and mortality are constantly increasing [1]. Non-small-cell lung cancer (NSCLC) accounts for 80–85% of all lung cancer cases. The National Comprehensive Cancer Network (NCCN) guidelines state that patients with stage Ia NSCLC do not require postoperative chemotherapy, while the patients with NSCLC in stages Ib, IIa, IIb, and IIIa require postoperative adjuvant chemotherapy to improve long-term survival [2].

In the 21st century, adjuvant chemotherapy is recommended for the postoperative management of stage Ib, II, and IIIa lung cancer patients as it improved the 5-year survival rate in several phase III clinical trials (ANITA and JBR10) and in a meta-analyses [3, 4]. The meta-analysis by the LACE Collaborative Group showed that surgery combined with cisplatin-based adjuvant chemotherapy significantly improved the overall survival compared with surgical treatment alone, and the 5-year absolute benefit in patients who underwent the chemotherapy was 5.4%. Therefore, the recommended standard adjuvant chemotherapy regimen for patients with NSCLC after complete surgical resection is the combined use of a platinum-based drug and a third-generation antitumour drug (gemcitabine, paclitaxel, and vinorelbine) [5].

Sensitivity to chemotherapy, both in the metastatic and in the adjuvant setting, differs by the clinicopathologic characteristics of patients [6–8]. Besides that, also molecular differences might be involved. As our understanding of the mechanism of chemotherapy sensitivity has advanced, it has been found that the changes in some molecular tumour markers may be related to the chemotherapy sensitivity. Therefore, in recent years, the concept of individualized therapy has taken hold. The selection of high-efficiency, less-toxic individualized chemotherapy regimens based on the expression levels of molecular markers in lung cancer tissues will be valuable in improving survival and quality of life in patients with NSCLC [9, 10].

Ribonucleotide reductase regulatory subunit 1 (RRM1) is often used as a predictive marker of gemcitabine efficacy [11, 12]. Clinical studies showed that gemcitabine had better efficacy in lung cancer patients with low RRM1 mRNA expression levels and prolonged their median survival time [13]. Tubulin beta 3 (TUBB3) is closely related to the effects of antimicrotubule agents. The close relationship between the *TUBB*3 mRNA expression level and the resistance to antimicrotubule agents in chemotherapy, especially paclitaxel, has been confirmed in many tumour cell lines and clinical studies [14]. Therefore, *TUBB*3 may be used as a prognostic indicator. High expression of *TUBB*3 in NSCLC patients was associated with poor prognosis. NSCLC patients with low *TUBB*3 expression had a better response to paclitaxel and had longer median survival times. In contrast, patients with high *TUBB3* expression had poor efficacy of chemotherapy with antimicrotubule agents [15]. The gene expression level of excision repair cross-complementation group 1 (ERCC1) directly affects the overall process of DNA repair. ERCC1 is involved in the development of resistance to platinum-based chemotherapeutic agents, and its expression level is negatively correlated with the efficacy of platinum-based chemotherapy agents and survival time [16]. Detection of *ERCC*1 mRNA expression level before platinum-based chemotherapy can improve the treatment efficacy and the survival rate of patients. Therefore, *ERCC*1 gene expression can be used as a marker for monitoring cisplatin efficacy.

Individualized treatment is the ultimate direction of chemotherapy for NSCLC. Chemotherapy regimens based on the information of molecular markers in patients have better efficacy, a better safety profile, and lower costs and result in better quality of life [17]. As different races carry different genetic information, their treatment regimens and efficacy should be different, as has been recognized in molecular-targeted therapy for patients with *EGFR* mutations [18]. In this study, a prospective randomized controlled trial was conducted to measure the molecular markers (mRNA expression of *ERCC*1, *RRM*1, and *TUBB*3) in tumour tissue specimens from patients who needed adjuvant chemotherapy after surgery. Based on the detected gene expression levels, the appropriate first-line chemotherapy regimen was selected for chemotherapy and the efficacy of gene expression-based chemotherapy was evaluated.

## 2. Data and Methods

### 2.1. Source of the Specimen

All NSCLC tissue specimens were obtained during surgery, and their locations were all in the central tumour area (nonnecrotic area), as confirmed by histopathological examination. The specimens were fixed in 10% formalin solution and sent to Yishan Medical Laboratory (Guangzhou, China), an independent third party, for gene expression detection. All patients signed an informed consent form. This study was approved by the Ethics Review Board of the First Affiliated Hospital of the Army Medical University.

### 2.2. Clinical Data

Patients who were diagnosed with NSCLC at the Ib-IIIa stage in the Department of Thoracic Surgery in the First Affiliated Hospital of the Army Medical University from July 2014 to June 2017 were enrolled in this study, and all patients voluntarily participated and signed an informed consent form. The subjects were diagnosed with NSCLC by clinical manifestations, medical history, and pathological result and had no history of other malignancies or relevant antitumour treatments before enrolment. The histopathological classification was based on the standard formulated by World Health Organization in 1999, and staging was based on the criteria developed by the International Union Against Cancer in 2009. This study enrolled a total of 150 patients. However, more than 50% of them quit the study, so only 67 patients successfully completed the follow-up. According to the follow-up results, they were randomly assigned into an experimental group (gene detected group, 39 cases) and a control group (undetected group, 28 cases). The experimental group had 28 males and 11 females, with an average age of 54.74 years, including 20 cases of squamous cell carcinoma and 19 cases of adenocarcinoma. The control group had 23 males and five females, with an average age of 51.78 years. There were nine cases of squamous-cell carcinoma, 17 cases of adenocarcinoma, and two cases of other malignancies.

### 2.3. Treatment Method

#### 2.3.1. Random Grouping

Subjects who met the inclusion criteria and signed the informed consent form were randomly assigned to the experimental group or the control group at a 1 : 1 ratio according to the minimization method (a dynamic randomized algorithm) by a central randomization system (which was supervised by Chengdu Mingke Hongneng Clinical Research Co., Ltd., an independent third party). The control group was then randomly divided into a gemcitabine group and a paclitaxel group (2 : 1 : 1 randomization). After randomization of the subjects, the central randomization system immediately assigned each subject a unique number (i.e., a random number) via SMS or Internet.

#### 2.3.2. Experimental Group

**Figure 1:**
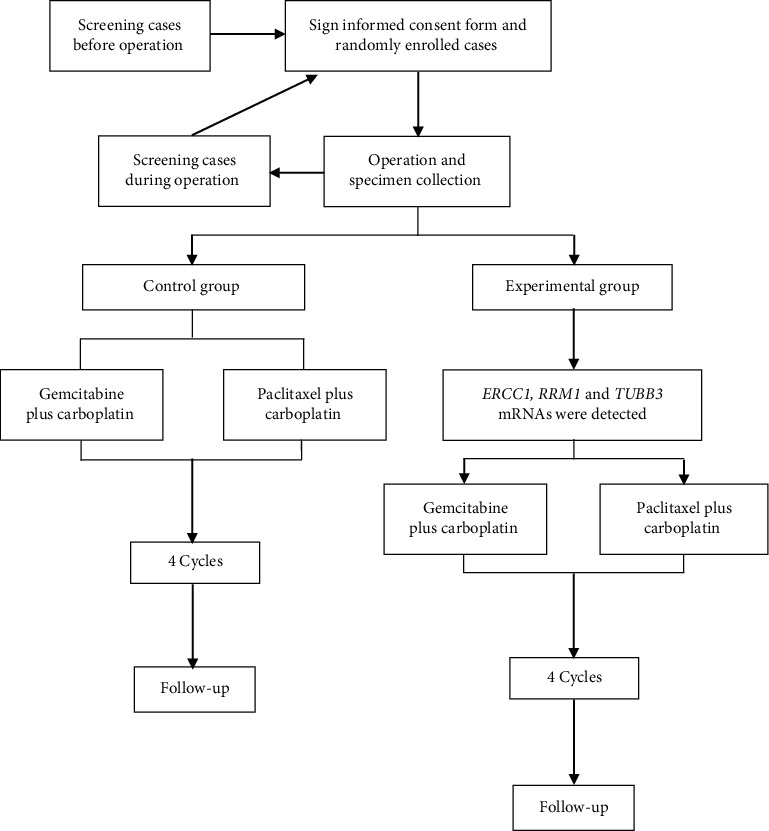
Experimental flowchart.

The platinum-based dual-agent chemotherapy regimen recommended by the NCCN guidelines was used for chemotherapy in NSCLC patients. Before chemotherapy, *ERCC*1, *RRM*1, and *TUBB*3 mRNAs were detected in patients in the experimental group to further divide them into a gemcitabine plus carboplatin group (ERCC1 <75%, RRM1 < TUBB3, and RRM1 < 75%) and a paclitaxel plus carboplatin group (ERCC1 < 75%, TUBB3 < RRM1, and TUBB3 < 75%) (Figure 1). The gemcitabine plus carboplatin group received four cycles (21 to 28 days per cycle) of chemotherapy with gemcitabine (1250 mg/m^2^, d1, 8) plus carboplatin (area under the curve (AUC) = 5, d1). The paclitaxel plus carboplatin group received four cycles (21 to 28 days per cycle) of chemotherapy with paclitaxel (1250 mg/m^2^, d1) plus carboplatin (AUC = 5, d1).

#### 2.3.3. Control Group

Patients randomized to the control group underwent four cycles (21 to 28 days per cycle) of chemotherapy with gemcitabine (1250 mg/m^2^, d1, 8) plus carboplatin (AUC = 5, d1) or four cycles (21 to 28 days per cycle) of chemotherapy with paclitaxel (1250 mg/m^2^, d1) plus carboplatin (AUC = 5, d1).

#### 2.3.4. Follow-Up

After the chemotherapy, patients were followed up once every 3 months in the first year and the second year and once every 6 months in the third year until the disease progressed or the 36-month follow-up was completed.

### 2.4. Method of Gene Expression Detection

The mRNA expression levels of *RRM*1, *TUBB*3, and *ERCC*1 were detected using the branched-DNA liquid chip technique. The specific steps were as follows. (1) An appropriate amount of the lysis buffer was added to formalin-fixed, paraffin-embedded samples, they were lysed at 56°C for 2 h, and the total mRNA purity in the lysis buffer was analysed. (2) The lysis buffer was transferred to the incubation plate, and the supportive probe-microspheres, supportive extension probes, and buffer were added and incubated at 55°C with shaking. (3) On the next day, the mixture was placed on a magnetic stand for 1 min. The supernatant was discarded. The wash solution was added, and the mixture was shaken for 1 min. After the mixture rested on the magnetic stand for 1 min, the supernatant was discarded. (4) The amplification and extension probes and the labelled probes were added to the wash solution at the same time, and the solution was shaken at 50°C for 1 h and placed on a magnetic stand for 1 min. The supernatant was discarded, and the mixture was rinsed with wash solution twice. (5) Streptavidin-phycoerythrin was added to the wash solution, followed by shaking at 50°C for 30 min. After the mixture rested on the magnetic stand for 1 min, the supernatant was discarded and the mixture was washed twice. Finally, the wash solution was added, with shaking for 5 min. (6) Data from a Luminex array reader were analysed to obtain the detected gene expression levels.

### 2.5. Patient Follow-Up

We conducted telephone or outpatient follow-up of patients with NSCLC enrolled in this study. The follow-up examinations included chest computed tomography, abdominal ultrasonography, cranial magnetic resonance imaging, whole-body bone scan, and positron-emission tomography-computed tomography if necessary. We defined postoperative recurrence and metastasis in lung cancer patients as the presence of extrapulmonary and intrapulmonary space-occupying lesions and typical lung cancer manifestations on imaging examinations. The follow-up lasted 3 years and ended on December 31, 2018.

### 2.6. Statistical Methods

Kaplan–Meier survival curves were drawn by R version 3.6.2 software to compare the disease-free survival (DFS: the length of time after treatment in which tumour is not detectable in patient's body) of the control group and experimental group. Univariate analysis for prognosis and the calculation of hazard ratios (HR) with corresponding 95% confidence intervals (CI) and logrank-*P* value were conducted using GraphPad Prism 7.0 to compare the efficacy and survival between the two groups of patients. Multivariate Cox regression analysis and Fisher's exact test were performed using the SPSS statistical software, version 19.0. *P* < 0.05 was considered significant.

## 3. Results

Univariate analysis of the prognosis of the enrolled patients showed that, between the two groups, there was no significant difference in the correlations between disease-free survival and age, sex, pathological type, stage, and gene expression detection (*P* > 0.05) (Table 1). Moreover, we compared the baseline characteristics of all patients. Fisher's exact test indicated that there was no significant difference in all clinicopathologic variable or treatment method (Table S1).

**Figure 2:**
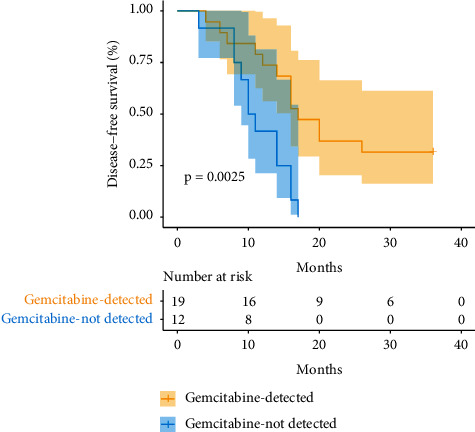
Survival analysis of patients' gene expression detected or not in the gemcitabine treatment subgroup.

The patients were divided into subgroups according to the tumour type, stage, age, years, sex, whether to detect the expression of three genes, and the treatment. The impact of gene expression detection on prognosis was analysed. The results suggested that, in the subgroups treated with gemcitabine, the median DFS was 17 months in the detected group and 10.5 months in the undetected group (hazard ratio (HR) = 0.2147, 95% confidence interval (CI): 0.07909–0.5827). That is, the risk of recurrence after gemcitabine treatment in the detected group was 0.2147 times that in the undetected group (Table 2). The survival curve is shown in Figure 2. The prognosis-related multivariate regression analysis showed no correlation between pathological type, stage, age, and sex in gemcitabine-treated patients (*P* > 0.05), but gene expression detection was independently correlated with DFS (*P*=0.025, 95% CI: 0.121–0.870) (Table 3).

**Figure 3:**
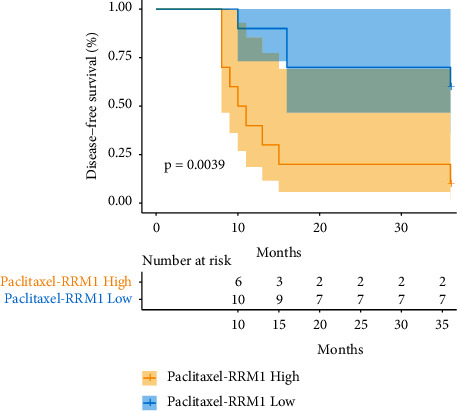
Survival analysis of patients with low *RRM*1 expression and patients with high *RRM*1 expression in the paclitaxel-treated subgroup.

Patients were dichotomized based on the median mRNA expression of *RRM*1, *TUBB*3, and *ERCC*1 genes. Patients with low *RRM*1 expression had a better prognosis after paclitaxel treatment than those with high *RRM*1 expression. The *χ*^2^ value of the logrank test was 8.350 (*P*=0.0039, HR = 0.1638, 95% CI: 0.04801–0.5588). The survival curve is shown in Figure 3.

## 4. Discussion

NSCLC is the most common lung cancer, accounting for approximately 80% of all lung cancer cases [19, 20]. The overall prognosis of NSCLC patients is poor due to the frequent occurrence of chemoresistance [21]. The expression of DNA repair-related genes in lung cancer cells is closely related to their chemotherapy resistance [22, 23]. Among these genes, *ERCC*1 is the most studied. Its low expression is often accompanied by an increase in the incidence of lung cancer, while its high expression can cause the rapid repair of damaged DNA in cells arrested at G2/M phase, resulting in cisplatin resistance [24]. RRM1 is a rate-limiting enzyme in the DNA synthesis pathway, and its high expression is associated with gemcitabine resistance [10]. TUBB3 protein, encoded by the *TUBB*3 gene, has the closest relationship with the sensitivity of cancer cells to antimicrotubule chemotherapeutic agents. Tumour patients with low expression of *TUBB*3 have a better response to paclitaxel and have longer median survival times, while the efficacy of antimicrotubule agents is poor for patients with high *TUBB*3 expression [25, 26]. Different individualized chemotherapy regimens targeting different molecular markers have had success in improving the survival time of NSCLC patients. Predicting the efficacy of chemotherapy by detecting certain molecular markers may be a way to improve the effectiveness of chemotherapy and the long-term survival rate of NSCLC patients in the future [27].

On the basis of experimental research, this study detected molecular markers (*ERCC*1, *RRM*1, and *TUBB*3 mRNAs) in tumour tissue specimens from patients who needed adjuvant chemotherapy after surgery. Based on the detected gene expression levels, the appropriate first-line chemotherapy regimen was selected. After four cycles of chemotherapy, patients were strictly followed up, and the DFS of each group of patients was statistically analysed. The relationship between the gene expression levels of *ERCC*1, *RRM*1, and *TUBB*3 and the sensitivity to first-line chemotherapy after surgery for NSCLC was prospectively investigated to find out whether *ERCC*1, *RRM*1, and *TUBB*3 genes could be used as markers for sensitivity to first-line chemotherapy. This study provides a theoretical and practical basis for postoperative individualized adjuvant chemotherapy for Chinese NSCLC patients and has important implications for both scientific research and clinical treatment.

A total of 67 patients who were enrolled in this study and had complete follow-up data were randomly divided into the experimental group (detected group) and the control group (undetected group) using the central randomization system. Since the small number of cases may have affected the correlation between gene expression detection and DFS, the number of cases needs to be expanded to verify our conclusions. In the subgroups treated with gemcitabine, gene expression detection was independently correlated with DFS after postoperative chemotherapy (*P*=0.025, 95% CI: 0.121–0.870), and the detection of expression of the *RRM*1, *TUBB*3, and *ERCC*1 genes reduced the risk of postoperative recurrence (*P*=0.0025, HR = 0.2147, 95% CI: 0.07909–0.5827), mainly because the gene expression results let patients with high *RRM*1 expression avoid gemcitabine treatment. The detection of *RRM*1 expression can help determine whether gemcitabine should be included in chemotherapy. Patients with high *RRM*1 expression were not suitable for gemcitabine chemotherapy, in line with a previous report [10]. Among the patients who underwent gene expression detection, patients with low expression of *TUBB*3 and *RRM*1 benefited most from paclitaxel treatment (*P*=0.0039, HR = 0.1638, 95% CI: 0.04801–0.5588). Detection of *TUBB*3 and *RRM*1 expression can help determine whether paclitaxel should be included in chemotherapy, and patients with low expression of *TUBB*3 and *RRM*1 are suitable for paclitaxel treatment [24]. Because of the high percentage of patients who quit this study, the targeted overall survival could not be reached. Therefore, more cases are needed and the follow-up mechanism needs to be improved.

In summary, the intratumoural expression levels of the three genes, *ERCC*1, *TUBB*3, and *RRM*1, were detected. Basing the chemotherapy regimens on the detected gene expression levels played a positive role in the control of disease progression. This study provides a basis for the clinical application of discoveries on chemotherapy resistance and new ideas for chemotherapy regimens in lung cancer. However, the occurrence and development of lung cancer are a long-term process with changes in multiple genes, and it is impossible for a single mechanism to completely explain chemotherapy resistance in lung cancer. To translate the discoveries on chemotherapy resistance into clinical practice, more prospective randomized clinical studies are needed to confirm the feasibility of the selection of chemotherapy regimens to ultimately improve the therapeutic efficacy and patient survival.

## Figures and Tables

**Table 1:** Univariate analysis of the prognosis of the enrolled patients.

Clinicopathologic variable	*N* = 67	Median DFS (months)	HR	95% CI	*P* value
*Tumour type*	Patient group				
Squamous cell carcinoma	29	16.00	0.6841	0.3754–1.247	0.2151
Adenocarcinoma	36	14.00			
Others (not included in statistical analysis)	2				
*Stage*					
I	28	20.00			
II	20	16.00			0.5412
III	19	14.00			
*Age*, *years*					
>52	32	16.50	0.8897	0.4925–1.607	0.6984
≤52	35	16.00			
*Sex*					
Male	51	16.00	1.096	0.5540–2.169	0.7918
Female	16	20.00			
*Whether to detect the expression of three genes*					
Yes	39	16.00	0.6693	0.3616–1.239	0.2011
No	28	12.50			
*Treatment*					
Gemcitabine	31	16.00	1.540	0.8447–2.806	0.1588
Paclitaxel	36	22.5			

**Table 2:** The patients were divided into subgroups according to their clinical characteristics, and the impact of gene expression detection on prognosis was analysed.

Variable	Patient group Experimental group vs. control group	Median DFS (months) Experimental group vs. control group	HR	95% CI	*P* value
*Tumour type*					
Squamous cell carcinoma	*n* = 19 vs. *n* = 17	16.00 vs. 14.00	0.7601	0.3518–1.642	0.4851
Adenocarcinoma	*n* = 20 vs. *n* = 9	21.00 vs. 10.00	0.5940	0.1951–1.808	0.3591
*Stage*					
I	*n* = 16 vs. *n* = 12	36.00 vs. 12.00	0.4111	0.1468–1.151	0.0905
II	*n* = 12 vs. *n* = 8	16.00 vs. 16.50	1.404	0.4515–4.366	0.5577
III	*n* = 11 vs. *n* = 8	16.00 vs. 11.00	0.5853	0.1939–1.767	0.3419
*Age*, *years*					
≤52	*n* = 19 vs. *n* = 16	16.00 vs. 10.5	0.5402	0.2313–1.262	0.1547
>52	*n* = 20 vs. *n* = 12	18.50 vs. 14.00	0.9117	0.3695–2.249	0.8409
*Sex*					
Male	*n* = 28 vs. *n* = 23	16.00 vs. 11.00	0.5825	0.2882–1.177	0.1323
Female	*n* = 11 vs. *n* = 5	20.00 vs. 22.00	1.127	0.2981–4.258	0.8605
*Treatment*					
Gemcitabine	*n* = 19 vs. *n* = 12	17.00 vs. 10.50	0.2147	0.07909–0.5827	0.0025
Paclitaxel	*n* = 20 vs. *n* = 16	16.00 vs. 22.50	1.041	0.4371–2.479	0.9279

**Table 3:** Multivariable Cox regression analysis of the prognosis of the patients treated with gemcitabine.

Clinicopathologic variable	*N* = 31	Median DFS (months)	HR	95% CI	*P* value
*Tumour type*					
Adenocarcinoma	21	14.00	0.895	0.344–2.327	0.819
Squamous cell carcinoma	10	16.00			
*Stage*					
I	12	16.00			0.281
II	7	16.00	0.508	0.188–1.377	0.183
III	12	11.00	0.459	0.149–1.420	0.177
*Age*, *years*					
>52	14	14.00	0.861	0.359–2.065	0.738
≤52	17	16.00			
*Sex*					
Female	5	20.00	1.518	0.364–6.340	0.567
Male	26	14.00			
*Whether to detect the expression of three genes*					
Yes	19	17.00	0.324	0.121–0.870	0.025
No	12	10.00			

## Data Availability

All data generated or analysed during this study are included in this published article.

## Supplementary Materials

Supplementary Materials Table S1: baseline characteristics of the enrolled patients.
