# Supplementary material for: Prospective Clinical Study of Postoperative Individualized Adjuvant Chemotherapy for Patients with Non-Small-Cell Lung Cancer Based on mRNA Expression of the Molecular Markers RRM1, TUBB3, and ERCC1
Source: J Oncol. 2021 Sep 23;2021:8820691. doi: 10.1155/2021/8820691 (PMC8486509; doi:10.1155/2021/8820691)
Supplement: Supplementary Materials — Table S1: baseline characteristics of the enrolled patients. [file 8820691.f1.docx]

Table S1. Baseline characteristics of the enrolled patients.

| **Clinicopathologic Variable** | **（N=67）** | **Detected** | **Not detected** | **P Value**  **Fisher’s exact test** |
| --- | --- | --- | --- | --- |
| **Tumor type** |  |  |  |  |
| Squamous cell carcinoma | 29 | 20 | 9 | 0.117 |
| Adenocarcinoma | 36 | 19 | 17 |  |
| Others（Not included in statistical analysis） | 2 | 0 | 2 |  |
| **Stage** |  |  |  |  |
| I | 28 | 16 | 12 | 1.000 |
| II | 20 | 12 | 8 |  |
| III | 19 | 11 | 8 |  |
| **Age, years** |  |  |  |  |
| ≤52 | 35 | 19 | 16 | 0.621 |
| >52 | 32 | 20 | 12 |  |
| **Sex** |  |  |  |  |
| Male | 51 | 28 | 23 | 0.393 |
| Female | 16 | 11 | 5 |  |
| **Treatment** |  |  |  |  |
| Gemcitabine | 31 | 19 | 12 | 0.804 |
| Paclitaxel | 36 | 20 | 16 |  |
